# Prior‐knowledge treatment planning for volumetric arc therapy using feature‐based database mining

**DOI:** 10.1120/jacmp.v15i2.4596

**Published:** 2014-03-06

**Authors:** Eduard Schreibmann, Tim Fox

**Affiliations:** ^1^ Department of Radiation Oncology and Winship Cancer Institute of Emory University Atlanta GA USA

**Keywords:** automated treatment planning, database mining, shape matching, shape similarity

## Abstract

Treatment planning for volumetric arc therapy (VMAT) is a lengthy process that requires many rounds of optimizations to obtain the best treatment settings and optimization constraints for a given patient's geometry. We propose a feature‐selection search engine that explores previously treated cases of similar anatomy, returning the optimal plan configurations and attainable DVH constraints. Using an institutional database of 83 previously treated cases of prostate carcinoma treated with volumetric‐modulated arc therapy, the search procedure first finds the optimal isocenter position with an optimization procedure, then ranks the anatomical similarity as the mean distance between targets. For the best matching plan, the planning information is reformatted to the DICOM format and imported into the treatment planning system to suggest isocenter, arc directions, MLC patterns, and optimization constraints that can be used as starting points in the optimization process. The approach was tested to create prospective treatment plans based on anatomical features that match previously treated cases from the institution database. By starting from a near‐optimal solution and using previous optimization constraints, the best matching test only required simple optimization steps to further decrease target inhomogeneity, ultimately reducing time spend by the therapist in planning arcs' directions and lengths.

PACS number: 87.55.D‐, 87.55.de

## I. INTRODUCTION

Volumetric arc therapy is an inverse treatment planning process that optimizes the intensity modulated by a multileaf collimator distribution as delivered during rotational therapy. The optimization aims to find a solution that trades off between irradiating the tumor and sparing normal tissue according to the dose‐volume histogram (DVH) constraints. However, the attainable DVH objectives for a patient‐specific anatomy are not known before planning, resulting in a lengthy guessing process that involves many rounds of optimization to progressively readjust the optimization constraints. Achievable DVH depends primarily on the anatomical location of the tumor relative to critical organs, with the planner modifying the energy, arc orientations, and MLC configurations, together with the DVH constraints, until the plan is considered clinically acceptable. It is unknown if the plan is optimal on the given patient anatomy until a complex multiobjective optimization is performed^1^, ^2^, ^3^ to deduce the Pareto front, defining the family of truly optimal plans where the dose to one objective cannot be decreased without degrading another. Currently multiobjective optimization is available only as a research tool; thus, in clinical practice, information of the attainable dose‐volume histograms of a given patient's anatomy is unavailable.

Current treatment planning systems are built on complex database systems, but this information is rarely used in the treatment planning process. On the basis of that observation, we propose a clinical solution that reproduces a dosimetrist's experience by searching the information within the database to query and retrieve previous cases of similar anatomy. In this database mining approach, the patient's segmentation is used as an input, then compared to previously treated patients to retrieve previous solutions and achievable DVH constraints. The planner uses these comparisons as guidance for the treatment process. Thus the approach speeds up the planning process by starting the optimization from previous fluences and DVH constraints as obtained in similar anatomy.

Our effort is encouraged by recent reports showing that integrating a priori information into the treatment planning process is efficient in IMRT. Indeed, incorporation of prior knowledge in the treatment planning process^4^, ^5^ is an effective method to reduce the lengthy times^(6)^ associated with selecting optimal beam configurations^7^, ^8^ and dose volume constraints.^(9)^ Wu and colleagues^10^, ^11^ investigated the use of overlaps between critical structures and the planning volume for predicting achievable DVH. Similarly, Zhu et al.^(12)^ used the database of previous plans to derive a set of attainable DVHs that are grouped together by anatomical similarity and with principal component analysis (PCA) used on the DVH to quantify their noticeable features as a dose prediction tool for prostate cases treated with IMRT. Geometry‐based approaches that consider individual patient topology have been also used to create maps that describe a specific direction to possibly deliver a tumor the prescribed dose without intersecting critical organs.^13^, ^14^ When incorporated into the treatment planning process,^6^, ^7^, ^15^, ^16^ this a priori information improves planning times, as the algorithm guesses favorable beam settings from the geometrical location of critical structures relative to the PTV. This work generalizes this concept by using previously treated cases as a preplanning tool that suggests attainable DVHs and constraints to further minimize the treatment planning process.

The novelty and critical aspect of our endeavor is the metric used to find similar case in the database. The metric is a single scalar value that quantifies the degree of matching between two cases. The technical difficulty arises as it must capture and describe similar and relevant features fast enough to enable comparison to hundreds of cases in a few minutes. In the proposed approach, matching is judged by a geometrical measure comparing position, shape of target, and critical organs receiving relevant dose. In the following, we present the technical aspects of the metric construction and search procedure, while illustrating its application in daily clinical operations.

### II. Materials and Methods

**Figure 1:**
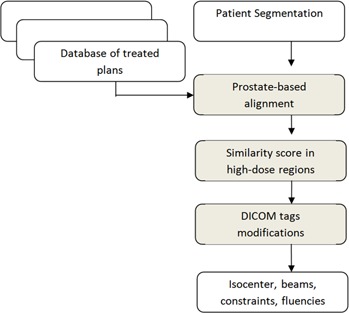
Flow chart of the proposed procedure: inputs (white rectangles) are a segmentation of the current patient and a database of previously treated cases; the algorithm's steps (gray rectangles) find the isocenter position by aligning the prostate in the patient and database plans using a rigid registration, then rank each plan using a geometrical overlap measure. For the best matching plan, planning information is reformatted to the DICOM format and can be imported in the treatment planning system to display arcs with their leaf positions and corrected isocenter position, as well as previously used optimization constraints and attainable DVHs.

A scheme of the overall procedure is presented in Fig. 1, detailing the input and algorithms used in our approach. First, important information from a treatment plan system database (Aria; Varian Medical Systems, Palo Alto, CA) is stripped of irrelevant information and saved in a custom format for easy access by the query procedure. When a new plan is to be generated, an interface tool allows the physician to specify the search criteria; then, cases within the search criteria are matched against the new case using a geometrical measure that compares the anatomy in the prostate and surrounding region receiving relevant dose. An optimization procedure is used in this process to iteratively move the isocenter until the surfaces to be compared are aligned; this is employed to align the new patient anatomy to the isocenter used in the database plan (see Materials and Methods section B). After spatial alignment, the similarity between the new plan and database plan is judged by a distance metric system, with the previous dosage plan used as region of interest to factor out irrelevant anatomy (Materials and Methods section C). By this procedure, the database plan with the closest geometrical resemblance to the new patient is identified. To automate the planning process, the corresponding arc settings and MLC positions are retrieved, reformatted, and saved as a new plan; it is then attached to the new patient scan so that it can be imported in the treatment planning system. If necessary, the dosimetrist can review, modify, and reoptimize the new plan (Materials and Methods section D).

### A. Database creation

The approach presented in the following discussion focuses on prostate carcinomas, a treatment site where the position and shape of target and critical structures varies little from patient to patient, which facilitates our endeavor. All clinical cases treated in our institution in the past two years since installation of volumetric arc therapy on our machines were identified and included in a specially formatted search database specifically designed and constructed for this project. This database is optimized for speed by including only the information needed for judging the geometrical matching of critical organs.

**Figure 2:**
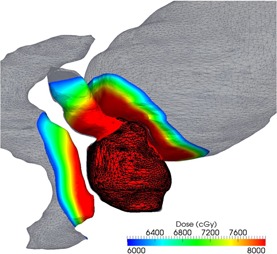
Information preprocessed from the database: the prostate, shown as a dark wireframe, is used to align the patient to the database cases. For improved similarity, calculation accuracy and geometric score are computed only in the high‐dose region, shown as a color‐coded overlay.

To construct this project‐specific database, a DICOM query tool was developed to query the treatment planning system patient database. The DICOM query tool inputs a list of patient IDs and extracts their corresponding images, segmentation, doses, and plans as plain DICOM files. As this extracted data include a significant amount of information irrelevant for this study, a cleaning‐up step eliminates unneeded files such as IMRT QA plans and datasets, treatment plans of the same patients for different sites, or diagnostic MRI scans that are not considered in this investigation. Upon cleaning, the remaining data are converted to a search‐friendly format that optimizes speed by cropping the CT scans and associated dose matrices for fast access; manipulation and structures names are automatically standardized. The typical information retained after this cleaning and reformatting process is shown in Fig. 2, where the whole anatomical information for a database case is shown as a gray wireframe, while the extracted geometry is shown as a solid surface color‐coded overlay with the dose delivered by the plan.

Concomitantly, a search index for advanced searches is created during this cleaning process. For each case, the index stores information such as the physician and institution, as well as treatment specific information such as the use of boost plans, number of arcs in the plans, and prescription dose. As treatment options and preferences vary between institutions and physicians, this nonanatomical information can be used as an option in the find procedure to filter the results by additional physician‐defined criteria.

### B. Target alignment

**Figure 3:**
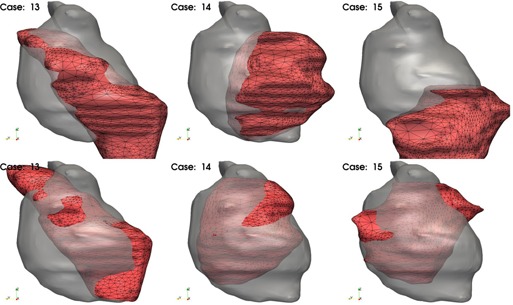
Alignment of patient and database plans for a series of three patients: upper row shows the segmentation of a patient's prostate as gray surface, and the database prostate as a red wired surface, before alignment; lower row shows the same display after the target is aligned in an iterative procedure that minimizes the distance between prostate shapes.

The prostate shape is the most important, as the high‐dose region has to match with this shape primordially. To compare the prostate shape in terms of the score function, we have to align it with the shape to which it is compared. This is realized by an iterative registration procedure that minimizes the distance between the points defining the patient and database surfaces. The algorithms starts by aligning the center of mass of the prostates, then iteratively modifies a transform that considers translations on all three axes to minimize the point‐by‐point mean distance between the aligned datasets. The procedure is illustrated in Fig. 3, where the upper row shows the surfaces before alignment, while the lower row shows the same surfaces after being aligned by the procedure.

### C. Search metric

**Figure 4:**
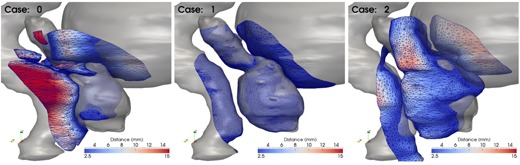
Plans scored by distance between surfaces in the high‐dose region and patient segmentations. The gray surface represents the patient segmentation, while the surface wireframe is the database segmentation in the high‐dose region color‐coded with the distance to the patient segmentation. The mean distance on this surface is used as similarity score.

Once the prostate is aligned, the degree of matching is quantified by lopping though all the points defining the geometry in the database plan, recording its closest distance to the new case plan, and computing the mean distance on all these points. The metric is computed on the surfaces of the prostate, bladder, and rectum. Note that the bladder and rectum are larger structures that may match near the prostate where the given dose is relevant, but may not match far away from the prostate in regions that receive little dose; thus, their shape in those far away regions is irrelevant for planning purposes. To increase the accuracy of the shape‐searching process, we crop these organs at risk to include only points receiving at least 80% of the prescribed dose, and compute the geometrical match only on these regions. The process is illustrated in Fig. 4, where surfaces cropped by the 80% isodose and used in the metric calculation are shown as a color‐coded surface. In the figure, color on this surface represents the distance to the new patient contours, shown as a grayscale surface.

## III. RESULTS

### A. Implementation

**Figure 5:**
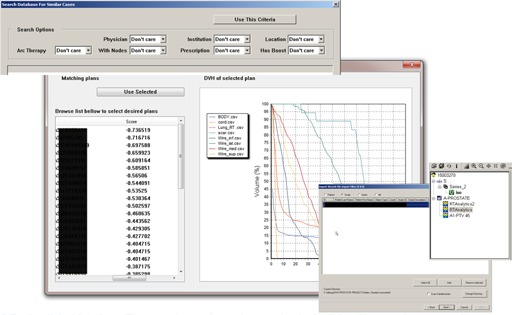
The tools interface.

The tools interface is presented in Fig. 5. A typical search would start with the user defining optional search criteria, such as the use of a boost plan or the restriction of a plans delivered by a specific accelerator or physician. The background window in the figure shows the results of a search query. The left list shows database matches, ordered by similarity with the most matching on top and decreasing towards the bottom. The physician can select a plan in the left box, and the corresponding DVHs will be displayed in the right panel. These previous DVHs can be used to suggest constraints based on previously achieved objectives on plans with similar anatomy. Once a plan is selected, the software tool modifies some relevant tags in the DICOM file by representing the previously used treatment plan and associating the information with the new patient. The original arc settings and MLC positions in the plan file are retained.

**Figure 6:**
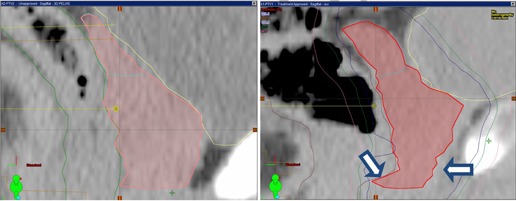
Sample matching result: the new patient CT dataset and prostate segmentation (left panel); the same display for the database case found to be the most similar (right panel). The general prostate shape is similar, with a few discordances visible at arrows. Target inhomogeneity caused by these discordances is solved by running the optimization procedure for a few iterations (shown in Fig. 7).

An example of the shape similarity found by the search module is shown in Fig. 6, where sagittal sections though the prostate center are shown for the new patient's geometric (left) and for the best matching plan in the database. The match will never be perfect; however, the aim here is to match the general shape of the target and have the delivered dose adapted to small geometrical changes by a few iterations of the optimizer. At the same time, DVHs constraints derived from the previous database case are used in the optimization as the general shape of the prostate and similar structures nearby. With this approach, and by having the matching database plan as a template to guide the optimization process, the planner does not have to guess the attainable DVH values. We call the following optimizations “mini‐optimizations”, as just a few iterations are used to correct the small inherent geometrical differences in prostate shape.

**Figure 7:**
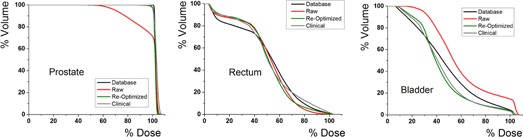
Comparison of prostate (a) rectum (b) and bladder (c) DVHs for the database plan (black line), raw best match plan without any optimization (red), same plan after a few iterations of the optimizer (green), and clinical plan used to treat the patient (gray). Few optimization iterations started from matched fluences and constraints are enough to bring the raw DVHs quality to clinical standards.

Comparison of the DVHs obtained before and after the mini‐optimization of the clinical plan used as part of the patient's standard care is presented in Fig. 7. The original DVHs of the database plan that best matched the new patient's anatomy on the original database segmentation is presented as black lines in the figure. The database plan just after import and the assignment to the new patient segmentation is shown as a red line. As seen for the prostate DVHs, the coverage is compromised due to these small geometric differences. Indeed, as observed also in Fig. 6, the new case prostate shape is larger at the arrows compared to the database plan, with the dose distribution not covering the target at these locations. The PTV coverage at 95% prescription dose is only 76.24% of the prostate volume. However, this drawback can be corrected by a few iterations of the optimizer from the database MLC position and with the use of previous arc configurations. In addition to the original fluences, we used constraints read from the database plan DVHs (shown also as black lines in Fig. 7), and upper constraints of 16% and 66% of the bladder volume not receiving more than 48 and 21 Gy, respectively, and for the rectum, 10% and 55% of its volume not receiving more than 66 and 42 Gy. The optimization was allowed to run for a maximum of 3 minutes or 20 iterations.

After this mini‐optimization, the PTV coverage increased to clinical levels at 99.1% of the volume for the 95% isodose line, as shown with a green line in Fig. 7. For comparison, the prostate's DVHs for the plan used to treat this case clinically is shown as a gray line in the figure, with the two graphs virtually indiscernible. For the same case, the dose distribution in critical structures improved to clinical standards, as shown for the rectum and bladder in the middle and right panels of the same figure. The plan, using knowledge from the database, was able to reach a DVH value of 6.5% at the 80% isodose, as compared to the clinical plan that spares the rectum at only 12.91%. For this organ, the use of prior knowledge improved sparing over the clinical plan that was created without any guidance. Similarly, the bladder's DVHs decreased after the mini‐optimization, to values similar to the clinical plan. Overall, the planning process was improved as the database solution eliminated the guesswork of selecting arcs lengths and directions, as well as critical structure constraints for the optimization procedure. Typically planning from scratch takes 1 to 2 hours, while with the settings suggested from previous plans a clinical acceptable plan was achieved in 15 minutes.

## IV. DISCUSSION

In the present report, we introduced an efficient method to search for previous cases with similar anatomy treated with volume arc therapy and suggested achievable DVH objectives that account for the trade‐offs between the target coverage and OAR sparing. This was done by using geometric and dosimetric information retrieved from a database of previous plans. Emphasis was placed on describing the search procedure and the associated metric to judge similarity between segmentations that match the patient's anatomy against a database of previously planned patients. As opposed to the standard approach of selecting settings in a trial and error process, in clinical practice, the proposed database search approach suggests settings that were found optimal in cases of similar anatomy.

Our approach is complementary to class solutions for prostate IMRT therapy to improve the speed by using a template of beam directions that are preoptimized. These class solutions are obtained by optimizing a set of cases and analyzing the beam directions to find a set of angles that are most probable to give an optimal dose distribution. The concept was first applied to conformal radiotherapy, as the standard four‐box technique is an established class solution, and was then extended to IMRT for different number of angles. As compared to the class solution, the technique proposed here does not generalize and, thus, does not lose details. The class solution does generalize by finding the directions that are common to a set of plans created for different patients. The database search engine presented here creates an automated plan that is, however, customized by taking into account individual segmentation details.

Current configuration uses a rather small database of only 83 patients to find the match, with larger databases being able to provide a better match at the expense of longer search times. Currently, a single comparison between surfaces takes 1 to 2 seconds on a standard desktop computer. Further analysis and daily clinical usage will establish the trade‐off between having larger databases on which a better match can be found, leading to potentially smaller reoptimization iterations, versus having smaller databases where a suboptimal match coupled with longer optimization iterations is preferred for practical considerations in a trade‐off between speed and accuracy.

## V. CONCLUSIONS

The approach improves the planning process for volumetric arc therapy by suggesting irradiation settings and attainable constraints as matched from prior planning solutions. This offers a method of predicting clinically achievable doses ahead of planning, and provides a starting optimization point for volumetric arc therapy by suggesting MLC leaf positions retrieved from previous planning solutions on patients with similar anatomy. The approach was illustrated for prostate cases and can be adapted in clinical practice with minor changes to the workflow.

## Supporting information

Supplementary Material
