# Supplementary material for: Prior‐knowledge treatment planning for volumetric arc therapy using feature‐based database mining
Source: J Appl Clin Med Phys. 2014 Mar 6;15(2):19–27. doi: 10.1120/jacmp.v15i2.4596 (PMC5875469; doi:10.1120/jacmp.v15i2.4596)
Supplement: Supplementary file 1 — Supplementary Material [file ACM2-15-19-s001.doc]

**Prior-Knowledge Treatment Planning for Volumetric Arc Therapy Using Feature-Based Database Mining**

Eduard Schreibmann c), Ph.D,

Tim Fox Ph.D.

Department of Radiation Oncology and Winship Cancer Institute of Emory University, Atlanta, Georgia

c) Author to whom correspondence should be addressed.

Winship Cancer Institute of Emory University

Department of Radiation Oncology

1316, Building A Emory Clinic

1365 Clifton Road NE

Atlanta, Georgia 30322

Tel 404.778.5667

Fax 404.778.4139
